# How supervisor support influences research innovation capability among master’s students: the chain mediating roles of research resilience and research engagement

**DOI:** 10.3389/fpsyg.2026.1929651

**Published:** 2026-09-01

**Authors:** Fei Wang, Liying Qi

**Affiliations:** 1 School of Management, Jiangsu University, Zhenjiang, Jiangsu, China; 2 School of Education, Jiangsu University, Zhenjiang, Jiangsu, China

**Keywords:** supervisor support, research resilience, research engagement, research innovation capability, master’s students

## Abstract

**Introduction:**

Cultivating innovative talent has become a prominent concern with the rapid advancement of artificial intelligence and intensification of knowledge production. Master’s students’ research innovation capability remains at a nascent stage. Previous studies have explored various factors that contribute to students’ research innovation, but few have investigated how resilience and engagement operate together in the sequential process.

**Methods:**

Grounded in Conservation of Resources theory, Social Support and Self-determination theory, this study developed and tested a chain mediation model to examine the mediating roles of research resilience and research engagement in the relationship between supervisor support and research innovation capability. Data were collected from 421 master’s students at Chinese universities. Path analysis and the bootstrap method were used to test the mediating effects.

**Results:**

Supervisor support exerted significant positive effects on research resilience (*β* = 0.521), engagement (*β* = 0.150). The direct effect of supervisor support on research innovation capability was non-significant, but the total effect was significant. Research resilience and engagement mediated the effect of supervisor support on research innovation capability (indirect effect = 0.156).

**Conclusion:**

This study revealed the psychological and behavioral mechanisms underlying the conversion of supervisor support into research innovation capability among master’s students. These findings underscore the importance of strengthening research resilience and fostering research engagement within master’s students training systems as key conditions for activating students’ research agency, which in turn enhances their research innovation capacity.

## 1 Introduction

The rapid advancement of artificial intelligence in higher education has accelerated technological change and intensified knowledge production (Wang H. et al., 2025). Innovation has emerged as a critical agenda across nations, and the cultivation of innovative talent has become a central concern in higher education worldwide. Higher education institutions increasingly emphasize research innovation, including original research contributions, complex problem-solving, and creative knowledge application (Zheng, 2023; Wong et al., 2025; Xing et al., 2026). In China, master’s programs are classified into two categories: academic master’s programs and professional master’s programs. Both types of programs emphasize the development of solid foundational theory, systematic specialized knowledge, innovative spirit, and innovative capacity as core training objectives. All master’s students are required to complete a thesis and undergo formal thesis evaluation. In universities, the master’s phase constitutes the foundational stage of the systematic development of research innovation capability. Furthermore, master’s students’ research innovation competence is inextricably linked to their potential for independent scholarly inquiry. However, at this stage, master’s students remain research “apprentices” who lack a comprehensive understanding of research norms and have limited capability to conduct research independently. Thus, they require adequate external support and resources. Moreover, pursuing research innovation is inherently challenging. Intractable problems abound, experimental setbacks are commonplace, and manuscript rejections are a recurring reality. Therefore, research innovation should not be framed as a purely intellectual endeavor. Developing research innovation capability not only hinges on external resources but also places considerable demands on master’s students’ psychological attributes and behavioral engagement in research.

Prior research has examined factors that influence postgraduate students’ research innovation capability from multiple perspectives. Some studies have focused on external environmental factors, identifying supervisor–student relationships, research climate, and peer support as significant predictors of research innovation capability (Zhang et al., 2023; Han et al., 2023; Becker et al., 2023; Ringo, 2025). Others have examined individual-level factors, finding that personality traits, academic interest, and intrinsic motivation also help shape postgraduate students’ innovation capacity (Zhang et al., 2023; Liu et al., 2023; Hu et al., 2025). Existing research focused on doctoral students have provided a preliminary foundation for understanding research innovation capability among master’s students, but empirical investigation into the influencing mechanisms of master’s students’ research innovation capability remains limited (Hu et al., 2025). In limited research, several areas warrant further exploration. First, while existing studies have examined the mechanisms through which supervisor support facilitates research innovation (Wang et al., 2026), comparatively less attention has been devoted to explicating the role of students’ own agency in the innovation process, specifically, how students actively appropriate and internalize supervisory resources rather than passively receiving them. Second, although prior research has investigated the contributions of both external environmental factors and internal individual factors to research innovation, these two categories of predictors have predominantly been treated as parallel pathways operating independently (Li S. et al., 2025). The transmissive process through which external support becomes internalized into students’ psychological mechanisms and behavioral patterns remains insufficiently elucidated. Third, existing studies have frequently positioned relatively stable variables (such as personality traits, academic interest, and motivation) as key mediators linking antecedents to research innovation capability (Hu et al., 2025; Chen and Chen, 2024). However, the research innovation process is inherently dynamic and iterative in nature, which raises questions about the explanatory adequacy of static psychological resources for capturing innovation outcomes that unfold within evolving and uncertain research contexts. Finally, although the positive association between external support and research innovation capability has been documented (Li S. et al., 2025), the extent to which and the conditions under which external support shapes students’ psychological and behavioral functioning remain open questions that deserve further empirical scrutiny. This raises a critical question: How can supervisor support most effectively foster master’s students’ research innovation capability? Answering this question requires moving beyond whether supervisor support matters to understanding how it can be utilized to develop research innovation capability and what psychological and behavioral mechanisms underlie this process.

Conservation of Resources (COR) theory provides a framework for addressing this question, positing that individuals are primarily motivated to acquire, protect, and maintain resources (Hobfoll, 1989; Hobfoll, 2001). In practice, research innovation is a prolonged and setback-laden endeavor. Accordingly, this study introduces two factors: research resilience and research engagement. Research resilience is rooted in the broader concept of resilience, which was originally used to describe the capacity of ecosystems to recover from disturbances and maintain structural integrity (Holling, 1973). It was subsequently extended to the domain of individual psychological resilience (Fletcher and Sarkar, 2013), conceptualized as a multidimensional psychological capacity through which individuals integrate internal and external resources. Research resilience refers to the ability to recover, adapt, and achieve positive outcomes when confronted with research-related adversity or setbacks. Studies have frequently examined psychological resources, such as self-efficacy and academic interest, as mediators that influence individual behavioral outcomes (Chen and Chen, 2024; Du et al., 2020; DeCaro et al., 2025). However, research resilience more adequately captures an individual’s capacity for recovery, adaptation, and regeneration under prolonged and high-risk conditions than these other psychological resources, given the distinctive nature of research innovation. Furthermore, research innovation requires sustained resource investment from individuals. Studies have largely focused on learning engagement, defined as a behavioral state characterized by vigor, dedication, and absorption in learning activities (Yu and Wu, 2024; Yang and Xiang, 2024). By contrast, research engagement refers to the behavioral state in which individuals demonstrate vigor, sustained dedication, and deep absorption in research activities. Unlike the constructs of motivation or academic effort commonly addressed in prior studies, research engagement more strongly emphasizes sustained and immersive behavior in the research context.

Building on the above analysis, this study focused on master’s students and develops and tests a chain mediation model. Specifically, this study examined the association between supervisor support and research innovation capability, as well as the sequential mediating roles of research resilience and research engagement in this relationship. Drawing upon Conservation of Resources theory, Social Support theory, and Self-Determination theory, this study aimed to elucidate the psychological and behavioral mechanisms through which supervisor support translates into research innovation capability. Furthermore, by testing the sequential mediating pathway, this study sought to determine the extent to which and through what transmissive processes supervisor support is progressively converted into research innovation capability. The study hoped to provide both theoretical foundations and practical implications for enhancing research innovation capability among master’s students.

## 2 Theoretical background and hypotheses

### 2.1 Supervisor support and research innovation capability

According to Social Support theory (Cobb, 1976), emotional, informational, and instrumental support that individuals or groups receive from their social environment promotes a sense of belonging, competence, and satisfaction. These supports will further influence behavioral patterns and developmental trajectories. Within the research context, supervisor support constitutes direct and critical social support resource for master’s students, characterized by two functions: resource supplementation and stress buffering. According to COR theory, individuals are fundamentally motivated to obtain and protect resources to achieve favorable outcomes (Hobfoll, 2001). Individuals are more inclined to engage in creative activities when their resource reserves are sufficient. Conversely, when their resources are depleted, individuals experience stress, thereby inhibiting positive behavioral performance. Research innovation is an inherently long-term and resource-intensive endeavor. Individuals must mobilize substantial cognitive, emotional, and other resources to advance their research innovation capability. As the most direct and significant external resource available to master’s students, supervisor support serves to expand their resource repertoire, thereby facilitating the development of research innovation capability.

Studies have established that supervisor support is a multidimensional form of resource provision, primarily encompassing instrumental and emotional support (Tenenbaum et al., 2001; Li H. et al., 2025), and can shape students’ attitudes and behaviors (Li H. et al., 2025; Blanchard and Haccoun, 2019; Brown et al., 2020). Empirical evidence indicates that a supervisory mentoring style is associated with postgraduate students’ creativity (Gu et al., 2015). Recent research demonstrates that supervisor support positively influences postgraduate students’ innovative behavior (Han et al., 2022). The more supervisor support master’s students receive, the more likely they are to develop research innovation capability. Accordingly, the following hypothesis is proposed:

*H1*: Supervisor support positively affects master’s students’ research innovation capability.

### 2.2 Mediating role of research resilience

Research innovation is characterized by extended time horizons and high uncertainty, during which individuals inevitably encounter setbacks or bottlenecks. According to COR theory, when confronted with external pressures, individuals can mobilize psychological resources to avoid resource depletion and goal attainment (Hobfoll, 2001). Research resilience refers to the capacity to recover, adapt, and thrive in the face of research-related adversity or setbacks (Rudd et al., 2021). Its development relies on the nourishment of external resources while simultaneously reflecting individuals’ ability to mobilize existing resources when navigating research adversity. Studies have demonstrated that teacher support is a vital external resource that can alleviate students’ sense of isolation under stress (Morin, 2020) and enhance their sense of belonging and satisfaction (He et al., 2025), thereby laying the groundwork for fostering resilience. The more supervisor support master’s students perceive, the more likely they are to view research challenges as opportunities for growth and, in turn, develop research resilience.

Research resilience is conceptually rooted in the broader constructs of resilience and psychological resilience (Holling, 1973; Fletcher and Sarkar, 2013). In the study, research resilience is conceptualized as a psychological quality and a dynamic capacity that develops through the ongoing interaction between external and internal resources. Based on COR theory, research resilience is an internal resource-regulating capacity that enables individuals to sustain existing resources and prevent resource depletion. Setbacks are inevitable in the context of research innovation. More resilient individuals are better positioned to regulate negative emotions and integrate external and internal resources, thereby advancing innovation. By contrast, less resilient individuals are more likely to become entrenched in negative emotions and fail to prevent resource loss, thereby impeding innovation. Empirical evidence suggests that resilience is a psychological capacity that is closely associated with creative performance under adverse conditions (Azazz and Elshaer, 2022). Recent research has demonstrated that resilient students tend to exhibit better academic performance (Cai and Meng, 2025). More resilient master’s students may be more disposed to mobilize cognitive and emotional resources and maintain an open and exploratory mindset, which in turn creates conditions for innovation development. Accordingly, the following hypothesis is proposed:

*H2*: Research resilience mediates the relationship between supervisor support and research innovation capability.

### 2.3 Mediating role of research engagement

Research engagement is derived from the concept of work engagement, defined as a positive, fulfilling state characterized by vigor, dedication, and absorption (Schaufeli et al., 2002). In the study, research engagement is defined as a behavioral state in which individuals maintain sustained energy in research, experience a profound sense of research meaning and complete full cognitive absorption in the research tasks. According to COR theory, individuals are capable of acquiring, mobilizing, and investing existing resources to prevent their depletion and obtain new resources (Hobfoll, 2001). Supervisor support in cognitive and emotional domains stabilizes and expands individuals’ resource repertoire. As a critical form of external resource support, supervisor support provides a solid foundation for individuals to mobilize and invest their resources. Considering the high uncertainty inherent in research innovation, supervisor support may reduce master’s students’ perceived risk of sunk costs. Moreover, supervisor support can alleviate students’ emotional burden and strengthen their willingness to invest resources. Recent research indicates that teacher support positively influences students’ academic engagement and safety perceptions (Jiang and Zhang, 2026; Lenzi et al., 2017). Thus, supervisor support, as an available external resource, may positively influence master’s students’ engagement behavior.

Research innovation demands sustained commitment and continuous investment by individuals. According to COR theory, individuals who possess greater resources are more willing to mobilize and invest their existing resources toward goal attainment, generating a gain spiral (Hobfoll, 2001). Specifically, when individuals proactively invest cognitive, emotional, and other resources, they establish a foundation for sustained exploration and productive outcomes, thereby facilitating the development of research innovation capability. This indicates that when master’s students are fully immersed in their research, they can create the conditions for innovative thinking and outcomes to emerge, thereby fostering the development of research innovation capability. Accordingly, the following hypothesis is proposed:

*H3*: Research engagement mediates the relationship between supervisor support and research innovation capability.

### 2.4 Chain mediating roles of research resilience and engagement

Grounded in Self-determination theory (Ryan and Deci, 2000), when the social environment satisfies individuals’ basic psychological needs, it catalyzes the internalization of external resources into autonomous motivation, thereby activating self-regulated and agentic functioning. From the perspective of COR theory (Hobfoll, 2001; Hobfoll et al., 2018), the influence of supervisor support on individuals’ research innovation capability is realized through two pathways. First, supervisor support, as a critical external resource, expands individuals’ resource repertoire and reduces resource depletion, thereby fostering resilience and providing an impetus for innovation. Second, through external resource support, supervisors enrich individuals’ resource repertoire and facilitate research integration and investment, thereby driving the development of innovation capability. Research resilience represents the capacity to recover and regenerate in the face of research adversity and serves as a critical juncture at which external resources are transformed into internal motivation. According to COR theory, research resilience is a psychological resource that motivates individuals to prevent further resource depletion through resource investment behavior (Hobfoll et al., 2018; Luthans et al., 2007; Bardoel and Drago, 2021). Specifically, individuals with greater research resilience are more likely to proactively invest resources in navigating research adversity, whereas those with less research resilience are more likely to withdraw from adversity and disengage from resource investment. Furthermore, research innovation is inherently a long-term activity fraught with setbacks. Throughout this process, the more resources individuals invest, the more capable they become of engaging in active exploration and translating efforts into outcomes, thereby facilitating the development of research innovation capability.

Research has shown that teacher support enhances students’ self-efficacy and sense of security (Lenzi et al., 2017; Duan et al., 2024), which are prerequisites for the formation of research resilience. Empirical evidence indicates that individual resilience influences behavioral engagement (Yang et al., 2022). Recent research has demonstrated that behavioral engagement is a significant predictor of innovative behavior (Husin et al., 2025). Master’s students are better positioned to sustain research engagement when they have sufficient internal and external resources, which fosters research innovation capability. This process exemplifies the resource gain spiral posited by COR theory. Accordingly, the following hypothesis is proposed:

*H4*: Research resilience and research engagement sequentially mediate the relationship between supervisor support and research innovation capability.

**Figure 1:**
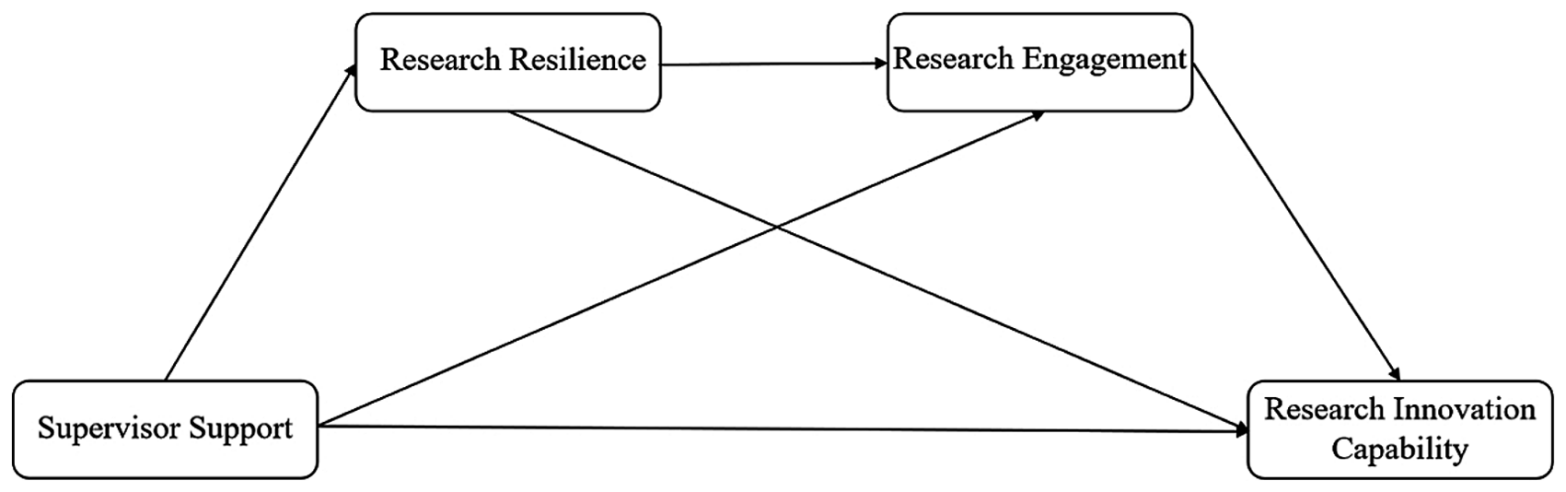
Theoretical model.

Based on the above analysis, the theoretical model is presented in Figure 1.

## 3 Method

### 3.1 Participants and data collection

Data were collected via an online survey administered between May 25 and June 21, 2025. The survey link was distributed to participants through the graduate school platforms of multiple Chinese universities. The questionnaire included a detailed introduction outlining the study’s purpose, assuring anonymity, and guaranteeing data confidentiality. IP address verification was implemented to ensure that each participant could submit only one response, and attention-check items were embedded throughout the questionnaire. All survey procedures adhered to the ethical principles of the Declaration of Helsinki. Completion and submission of the questionnaire were considered as implied consent to participate.

The respondents were enrolled master’s students from diverse disciplines across multiple Chinese universities. A total of 450 master’s students completed the questionnaire via the online platform. Responses were screened based on attention-check items, completion time, and response patterns to ensure data quality. Questionnaires with incorrect attention-check responses, excessively short completion times, or invariant response patterns (identical answers across all items) were excluded, resulting in the removal of 29 invalid responses. This yielded 421 valid responses, for an effective response rate of 93.56%. According to Kline, the minimum sample size for structural equation modeling should be no fewer than 200, and the ratio of the sample size to freely estimated parameters should be no less than 10:1 (Kline, 2016). The final valid sample of 421 respondents satisfied both criteria. Table 1 presents the demographic characteristics of the final sample.

**Table 1:** Demographic characteristics of the sample (*N* = 421).

Variable	Category	Frequency	Percentage
Gender	Male	178	42.30%
	Female	243	57.70%
Academic year	First year	133	31.60%
	Second year	145	34.40%
	Third year	143	34.00%
Institutional tier	Double first-class university	35	8.30%
	First-class discipline university	28	6.70%
	National key university	90	21.40%
	Provincial key university	116	27.60%
	Regular undergraduate institution	152	36.10%
Training orientation	Academic master’s	183	43.50%
	Professional master’s	238	56.50%
Disciplinary category	Humanities and social sciences	117	27.80%
	Natural sciences	304	72.20%

### 3.2 Measurements

All constructs were measured using established scales that were either adopted or adapted from prior research. All items were rated on a 5-point Likert scale ranging from 1 (not at all true) to 5 (completely true). All items underwent a translation and back-translation procedure to ensure linguistic equivalence (Brislin, 1970).

#### 3.2.1 Supervisor support

Supervisor support was measured using a scale adapted from Overall et al. (2011), encompassing three dimensions. The original scale was modified to suit the research context of master’s students, yielding nine items. Sample items included: “My supervisor provides me with research guidance,” “My supervisor comforts or encourages me when I encounter setbacks in research,” and “My supervisor encourages me to develop my own perspectives and ideas during the research process.” Higher scores indicated greater perceived supervisor support (Cronbach’s *α* = 0.969).

#### 3.2.2 Research resilience

Research resilience was measured using items drawn from the Connor-Davidson Resilience Scale (CD-RISC). The original 25-item CD-RISC, developed by Connor and Davidson (2003), comprises five dimensions: personal competence, trust in one’s instincts, positive acceptance of change, control, and spiritual influence. Campbell-Sills and Stein (2007) subsequently developed a 10-item abbreviated version (CD-RISC-10). Drawing on these established measures, this study adapted the scale to reflect the research context of master’s students, resulting in 10 items. Sample items included: “I am able to recover after experiencing setbacks or difficulties in research,” “When doing research, I can adapt to changes,” and “I am not easily discouraged by research failures.” Higher scores indicated stronger research resilience (Cronbach’s *α* = 0.970).

#### 3.2.3 Research engagement

Research engagement was measured using a scale adapted from the Utrecht Work Engagement Scale (UWES-9) developed by Schaufeli et al. (2006), which encompasses three dimensions. Research engagement represents a domain-specific form of work engagement; therefore, the scale was modified to reflect the characteristics of master’s students, yielding a total of nine items. Sample items included: “I feel energetic and full of vitality when conducting research.” “I can work on research continuously for a long time” and “When I focus on research, time flies by.” Higher scores indicated greater research engagement (Cronbach’s *α* = 0.964).

#### 3.2.4 Research innovation capability

Research innovation capability has been operationalized from multiple perspectives in the literature. Some scholars have focused on innovative personality (Gough, 1979), others have emphasized innovative behavior and practical output (Scott and Bruce, 1994), while others have assessed academic innovation through the lens of scholarly outcomes (Lin, 2016). Integrating prior research with the Chinese context, Ma and Yao (2024) developed a scale measuring postdoctoral research innovation capability comprising research innovative thinking, practice, personality and so on, consisting of 18 items. Following confirmatory factor analysis, three items with standardized factor loadings below the acceptable threshold were removed (Hair et al., 2019), resulting in 15 items. The revised model demonstrated satisfactory fit indices (*χ*^2^/d*f* ≈ 2.258, TLI = 0.987, CFI = 0.991, SRMR = 0.022, and RMSEA = 0.055). Sample items included “I possess a certain degree of divergent thinking in my research” and “I believe that conducting research requires a certain level of imagination” (Cronbach’s *α* = 0.972).

### 3.3 Data analysis

Data were analyzed using SPSS 25.0, Mplus 8.3 and SmartPLS 4.1.1.1. The reliability and validity of the measurement scales were first assessed using internal consistency coefficients, confirmatory factor analysis (CFA), average variance extracted (AVE), composite reliability (CR) and heterotrait-monotrait ratio of correlations (HTMT) (Hair et al., 2019; Hu and Bentler, 1999; Brown, 2015; Fornell and Larcker, 1981; Henseler et al., 2015). Common method bias (CMB) was examined using the unmeasured latent method construct (ULMC) approach (Williams et al., 2010). Descriptive statistics and bivariate correlations were then computed for all core variables. The path coefficients and bootstrap method with resamples (Shrout and Bolger, 2002; Preacher and Hayes, 2008; Hayes, 2013; Yu et al., 2025) was used to examine the mediating effects of research resilience and engagement on the relationship between supervisor support and research innovation capability. Gender, academic year, institutional tier, training orientation, and disciplinary category were included as covariates to control for potential confounders.

## 4 Results

### 4.1 Validity test and common method bias

CFA was performed using Mplus 8.3 to evaluate the construct validity of the hypothesized four-factor measurement model, comprising advisor support, research resilience, research engagement, and research creativity. Table 2 reports the fit indices for four alternative measurement models. Based on the criteria recommended by Hu and Bentler (Hu and Bentler, 1999), the hypothesized four-factor model yielded adequate fit: *χ*^2^/d*f* ≈ 2.258, TLI = 0.987, CFI = 0.991, SRMR = 0.022, and RMSEA = 0.055. Moreover, in accordance with the information criteria proposed by Akaike (1974) and Schwarz (1978), the four-factor model produced the smallest AIC (6353.367) and BIC (6523.157) values relative to all alternative models, confirming that the four constructs are empirically distinguishable, and the measurement model has adequate discriminant validity.

**Table 2:** Confirmatory factor analysis and model comparison (*N* = 421).

Model	χ^2^/d*f*	CFI	TLI	RMSEA	SRMR	AIC	BIC
Four-factor model	2.258	0.991	0.987	0.055	0.022	6353.367	6523.157
Three-factor model^a^	18.979	0.860	0.819	0.207	0.056	7206.895	7364.558
Two-factor model^b^	25.216	0.805	0.757	0.240	0.049	7571.454	7721.031
Single-factor model^c^	41.396	0.668	0.594	0.310	0.112	8468.361	8613.896

a Research resilience and engagement were combined into one factor.

b Research resilience, engagement, and innovation capability were combined into one factor.

c All observed indicators were combined into one factor.

***p < 0.001.

The partial least squares (PLS) algorithm in SmartPLS 4.1.1.1 was employed to evaluate the outer loadings of all measurement items. Results indicated that item loadings for the perceived supervisor support scale ranged from 0.849 to 0.932, research resilience from 0.845 to 0.916, research engagement from 0.749 to 0.941, and research innovation capability from 0.721 to 0.903. All outer loadings exceeded the recommended threshold of 0.70 (Hair et al., 2019). Following guidelines for assessing measurement quality (Hair et al., 2019; Fornell and Larcker, 1981), composite reliability (CR) values for all constructs surpassed 0.90, average variance extracted (AVE) values for all constructs exceeded 0.50, indicating satisfactory internal consistency and validity. According to guidelines (Henseler et al., 2015), the heterotrait-monotrait ratio of correlations (HTMT) values for all construct pairs ranged from 0.454 to 0.816 (below 0.85), the upper bounds of the bias-corrected 95% bootstrap confidence intervals for all HTMT values reached a maximum of 0.858 (below 1.0). These results confirmed adequate discriminant validity.

All variables were assessed via self-report by the same respondents; therefore, common method bias (CMB) was evaluated. Following Podsakoff et al. (2012), a single-factor CFA was conducted to gage CMB severity. The single-factor model demonstrated poor fit, with all indices falling below acceptable thresholds: *χ*^2^/d*f* ≈ 41.396, CFI = 0.668, TLI = 0.594, SRMR = 0.112, and RMSEA = 0.310. The hypothesized four-factor model fit the data significantly better than the single-factor model. To further mitigate the risk of common method bias, the unmeasured latent method construct (ULMC) approach was employed (Podsakoff et al., 2003). A five-factor model incorporating an unmeasured method factor was compared with the four-factor measurement model. The results revealed that the change in CFI (ΔCFI = 0.007) was below the critical threshold of 0.01 (Cheung and Rensvold, 2002), indicating that common method bias did not pose a substantial threat to the validity of the present findings.

### 4.2 Descriptive statistics and correlation analysis

The descriptive statistics reported in Table 3 provide a preliminary overview of the four core variables. Master’s students reported relatively high levels of supervisor support (*M* = 4.255), suggesting that supervisors offer substantial academic guidance, emotional support, and autonomy in research development. Research resilience (*M* = 3.810) and research engagement (*M* = 3.488) were also at favorable levels, indicating that master’s students demonstrated a reasonable degree of resilience and devoted considerable time and effort to their research activities. Research innovation capability (*M* = 3.750) fell in the upper-moderate range, suggesting that students had a certain capacity for innovation in the research process.

**Table 3:** Descriptive statistics and correlation analysis.

Variable	*M*	SD	1 (SS)	2 (RR)	3 (RE)	4 (RIC)
1. Supervisor support	4.255	0.839	1			
2. Research resilience	3.810	0.811	0.514***	1		
3. Research engagement	3.488	0.920	0.492***	0.765***	1	
4. Research innovation capability	3.750	0.730	0.440**	0.793***	0.788***	1

*p < 0.05; **p < 0.01; ***p < 0.001.

The bivariate correlations presented in Table 3 offer preliminary evidence of the hypothesized relationships among the variables. Supervisor support was significantly and positively correlated with research resilience (*r* = 0.514, *p* < 0.001), research engagement (*r* = 0.492, *p* < 0.001), and research innovation capability (*r* = 0.440, *p* < 0.001). Research resilience was also significantly and positively associated with research engagement (*r* = 0.765, *p* < 0.001) and research innovation capability (*r* = 0.793, *p* < 0.001). Research engagement was significantly and positively related to research innovation capability (*r* = 0.788, *p* < 0.001). These results provide preliminary support for the hypothesized model and warrant further examination through mediation analysis.

### 4.3 Chain mediation effect test

**Figure 2:**
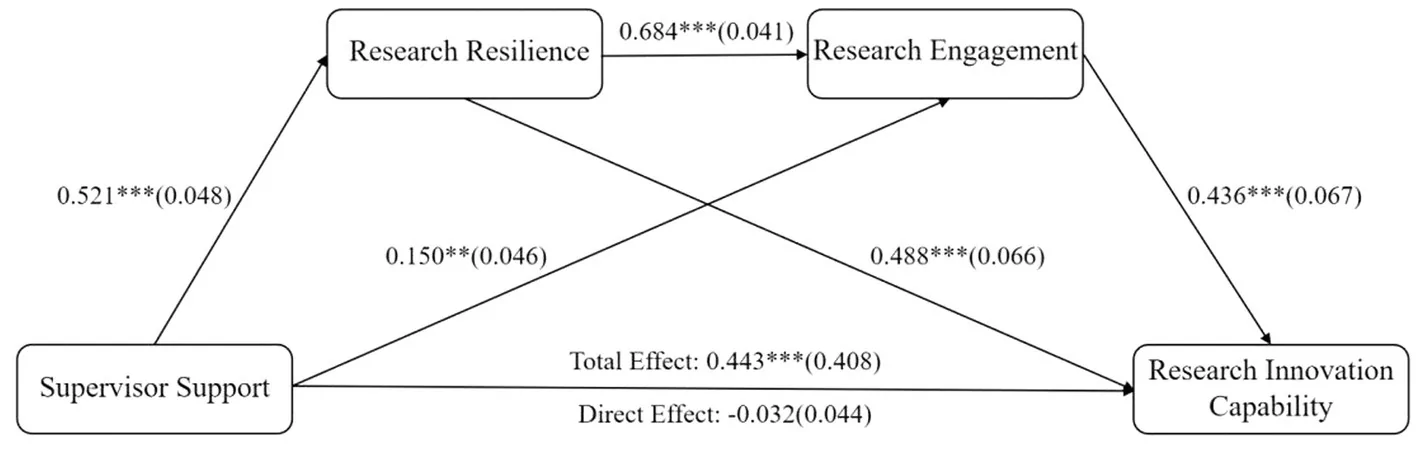
Path coefficients. *p < 0.05; **p < 0.01; ***p < 0.001.

As shown in Figure 2, the total effect of supervisor support on research innovation capability was significant (*β* = 0.443, *p* < 0.001), but the direct effect of supervisor support on research innovation capability became non-significant (*p* > 0.05). Supervisor support positively predicted research resilience (*β* = 0.521, *p* < 0.001), while research resilience positively predicted research innovation capability (*β* = 0.488, *p* < 0.001), thus supporting H2. Similarly, supervisor support significantly predicted research engagement (*β* = 0.150, *p* < 0.01), and research engagement significantly predicted research innovation capability (*β* = 0.436, *p* < 0.001), supporting H3. Furthermore, research resilience was a significant positive predictor of research engagement (*β* = 0.684, *p* < 0.001), providing preliminary support for H4.

To further verify the robustness of the mediation effects, bootstrapping with 5,000 resamples was employed, and the results are summarized in Table 4. The indirect effect of supervisor support on research innovation capability through research resilience was significant (indirect effect = 0.254, 95% CI [0.179, 0.344]), confirming that research resilience mediates the relationship between supervisor support and research innovation capability. Thus, H2 was further corroborated. The indirect effect through research engagement was also significant (indirect effect = 0.066, 95% CI [0.027, 0.114]), indicating that research engagement mediates the relationship between supervisor support and research innovation capability. Thus, H3 was supported again. The sequential indirect effect through research resilience and then research engagement was also significant (indirect effect = 0.156, 95% CI [0.099, 0.220]), demonstrating that research resilience and engagement jointly function as serial mediators in the relationship between supervisor support and research innovation capability, thereby supporting H4. Although the direct effect was −0.032 (95% CI [−0.125, 0.047]), the total effect was 0.443 (95% CI [0.349, 0.531]). Following the analytical framework outlined by Hayes (2017), these results indicate that the influence of supervisor support on research innovation capability is fully transmitted through the serial pathway of research resilience and engagement. Thus, H4 was conclusively supported.

**Table 4:** Summary of mediation effect sizes.

Path	Effect size	SE	95% CI		Outcome
			LLCI	ULCI	
Total effect	0.443	0.048	0.349	0.531	Significant
Direct effect	−0.032	0.044	−0.125	0.047	Non-significant
Supervisor support → research resilience → research innovation capability	0.254	0.042	0.179	0.344	Significant
Supervisor support → research engagement → research innovation capability	0.066	0.022	0.027	0.114	Significant
Supervisor support → research resilience → research engagement → research innovation capability	0.156	0.031	0.099	0.220	Significant

The study also examined the effect sizes (f^2^) for each structural path. According to guidelines for *f*^2^ (Hair et al., 2019), the results indicated that the effects of supervisor support on research resilience (*f*^2^ = 0.363) and research resilience on research engagement (*f*^2^ = 0.895) were both large. The effects of research resilience on research innovation (*f*^2^ = 0.287) and research engagement on research innovation (*f*^2^ = 0.266) were both medium. The effect of supervisor support on research engagement was 0.034. Notably, the direct effect of supervisor support on research innovation was negligible (*f*^2^ = 0.001), lending further support to the full chain mediation mechanism.

## 5 Discussion

### 5.1 Relationship between supervisor support and research innovation capability

Supervisor support is positively associated with master’s students’ research innovation capability, but this relationship operates primarily through indirect pathways. The findings indicated a significant positive association between supervisor support and research innovation capability, underscoring the importance of external support, consistent with social support theory (Cobb, 1976). Specifically, supervisors provide instrumental support by offering research platforms and methodological guidance, emotional support through patient mentoring that addresses affective needs, and autonomy support by encouraging independent inquiry that stimulates creative vitality. These findings align with prior evidence that supervisor support positively relates to individual competence and productive behaviors (Gu et al., 2015; Han et al., 2022). Notably, the direct effect of supervisor support on research innovation capability was non-significant. This suggests that supervisor support does not translate into innovation capability in an unmediated fashion but rather operates through intermediate psychological and behavioral processes. In other words, supervisors, as significant others, function as enablers rather than determinants of research innovation capability development.

### 5.2 Mediating role of research resilience between supervisor support and research innovation capability

Research resilience emerged as a significant mediator in the relationship between supervisor support and research innovation capability. And this indirect pathway accounted for a relatively large proportion of the total effect. This mediating mechanism reveals two characteristics of supervisor support: it furnishes new external resources for master’s students, consistent with prior findings (Ringo, 2025; Cai et al., 2026), and it awakens students’ sense of research agency. Moreover, research resilience, as a psychological resource, plays a more critical role in the conversion of external resources. Setbacks and obstacles are inevitable in the research process. Supervisors’ academic guidance reduces resource depletion stemming from cognitive overload, while their emotional support alleviates feelings of insecurity and prevents the erosion of psychological resources. Through these protective mechanisms, master’s students progressively develop research resilience and become willing to confront novel challenges, thereby generating sustained motivation for research innovation.

### 5.3 Mediating role of research engagement between supervisor support and research innovation capability

Research resilience and research engagement sequentially mediated the relationship between supervisor support and research innovation capability, constituting a chain mediation pathway. This finding highlights the importance of master’s students exercising research agency, consistent with self-determination theory (Ryan and Deci, 2000). Critically, research resilience demonstrated a stronger mediating role than research engagement in this process, indicating that resilience serves as a pivotal hub through which master’s students convert external resources into internal growth amid the demands of sustained research innovation. The chain mediation further reveals that supervisor support, as an external resource, constitutes the starting point for promoting research innovation capability, yet this external support achieves positive transformation only through students’ internal psychological and behavioral processing. Within this transmission chain, research resilience serves a resource protection and restoration function, whereas research engagement fulfills a resource conversion function. Collectively, this serial mediation elucidates the transmission mechanism through which supervisor support progressively fosters the development of research innovation capability among master’s students.

### 5.4 Chain mediating roles of research resilience and research engagement in the relationship between supervisor support and research innovation capability

These findings demonstrate that supervisor support enhances research resilience among master’s students, which in turn stimulates greater research engagement, ultimately fostering the development of research innovation capability. Research resilience and engagement were serial mediators in the relationship between supervisor support and research innovation capability. Supervisor support is a critical external resource in the research process that constitutes the starting point of the chain through which research innovation capability is cultivated. From the perspective of COR theory, supervisor support is an external resource that alleviates research-related stress and facilitates the accumulation of psychological resources. Master’s students gradually develop research resilience as these psychological resources are progressively enriched. Students with greater research resilience are better positioned to conserve, regenerate, and reinvest resources, thereby creating favorable conditions for the development of research innovation capability. Within this process, research resilience has a protective and restorative function with respect to resources, whereas research engagement operates as a mechanism for activating and converting those resources into productive scholarly action. Notably, as an external resource, supervisor support can maximally promote research innovation capability only when it is internalized as psychological capital and subsequently translated into active resource investment. Thus, this chain mediation pathway elucidates the transmission chain through which supervisor support fosters research innovation capability among master’s students.

## 6 Conclusion and implications

### 6.1 Conclusion

Grounded in COR theory, this study developed and tested a chain mediation model to examine the underlying mechanisms linking supervisor support to research innovation capability among master’s students. In this model, supervisor support was conceptualized as an external resource, whereas research resilience and engagement were specified as sequential mediators. Data were collected from 421 master’s students across multiple universities in China. The results supported the hypothesized model and yielded several findings with implications for promoting research innovation capability among master’s students. And the study underscores the importance of strengthening research resilience and fostering research engagement within master’s students training systems as key conditions for activating students’ research agency, which in turn enhances their research innovation capacity.

### 6.2 Theoretical implications

This study provides several theoretical contributions. First, it extends COR theory, Social Support and Self-determination theory to a new context. By focusing on the master’s student population, this study examined the process through which external support is converted into capability, demonstrating the theories in explaining competence development among graduate students. Second, although prior research has predominantly applied COR theory to investigate the detrimental effects of stress (Smith et al., 2025; Xin et al., 2025), this study empirically supports the positive logic of the theory that external resources can be internalized as psychological resources and translated into active resource investment, ultimately yielding positive outcomes. Third, this study contributes to the growing body of literature on academic resilience. Prior research has primarily examined the relationship between academic resilience and negative outcomes, such as burnout (Emerson et al., 2023). However, this study views research resilience and engagement as critical points of conversion through which supervisor support is channeled. This offers a more nuanced explanation of how research innovation capability is fostered among master’s students. Fourth, this study enriches the literature on the development of research innovation capability in graduate education. Prior research has largely focused on innovative competence or performance among undergraduate populations (Zhu and Zhang, 2011; Liu et al., 2024). By contrast, this study specifically targets master’s students, whose research innovation capability is at a nascent stage and thus warrants dedicated scholarly attention.

### 6.3 Practical implications

First, universities should strengthen their supervisory support systems and optimize the allocation of mentoring resources. Research innovation is inherently characterized by extended timelines and normalization of setbacks. Master’s students are in the initial stage of their research trajectories, and their research innovation capability is in a nascent phase. Therefore, supervisor support is essential for establishing a solid foundation for its development. University administrators should build comprehensive supervisory support platforms that diversify mentoring resources and refine the structure of supervisory provision. Institutions could consider establishing dedicated mentoring centers for master’s students that integrate research guidance with emotional support. Furthermore, universities should encourage the adoption of co-supervision models, such as pairing principal and associate supervisors or combining senior and early-career faculty members, to enhance the collective capacity to support research innovation among master’s students.

Second, supervisors should provide adequate instrumental and emotional support. Supervisors are the most proximate external resource available to master’s students and thus represent a vital force for cultivating research innovation. Supervisors should design well-structured support strategies, including assisting students in developing research plans, maintaining a consistent rhythm of academic mentoring, and facilitating access to high-quality research resources. In addition to research-oriented guidance, supervisors should attend to their students’ emotional needs by minimizing dismissive or discouraging communication and instead providing constructive and affirming feedback. Supervisors are also encouraged to foster a research community with their students, thereby nurturing a sense of shared scholarly purpose. Furthermore, while offering thorough guidance, supervisors should allow sufficient space for students’ autonomous exploration to sustain a balanced approach that is neither overly directive nor excessively permissive.

Third, master’s students should proactively transform the external resource of supervisor support into research resilience and sustained research action. As the principal agents of the research process, master’s students are the primary actors driving research innovation. Competition in scholarly research has intensified considerably with the rapid advancement of artificial intelligence and technology (Wang C. et al., 2025; Jing et al., 2024). However, supervisor support functions in a facilitative capacity, empowering the development of research innovation capability rather than replacing the student’s agency in scholarly inquiry. As Max Weber articulated through his concept of “science as a vocation” (Frommer and Frommer, 2010), the pursuit of scholarly innovation is fundamentally a personal commitment, and robust research resilience coupled with sustained engagement represents the enactment of such agency. Accordingly, master’s students should not rely exclusively on supervisor support but instead actively internalize these external resources as intrinsic motivation, fortifying their resilience and deepening their engagement to ultimately advance the development of research innovation capability.

In conclusion, this study offers recommendations across institutional, supervisory, and individual levels. This also provides empirical evidence that may inform the design and refinement of research-oriented postgraduate training systems, such as Mphil (Albejaidi and Mughal, 2026).

### 6.4 Limitations

This study had some limitations. First, regarding sample representativeness, the data were drawn exclusively from master’s students enrolled in Chinese universities, and the cross-cultural generalizability of the findings remains to be established. Future research should replicate this model across diverse cultural and institutional contexts to enhance its external validity. Second, all variables were assessed using self-report measures, which may be susceptible to subjectivity and CMB. Although self-report instruments are widely used in this type of research, this measurement approach has inherent limitations. Future studies should adopt triangulation methods to strengthen the robustness of these findings. Third, the cross-sectional nature of the data collection imposes constraints on causal inference. Future research would benefit from multi-source data or longitudinal designs. Fourth, the development of research innovation capability among master’s students may be influenced by additional organizational factors that were not examined in this study. Future research could explore multilevel analysis or experimental study to achieve a more comprehensive understanding of the pathways through which research innovation capability is fostered.

## Data Availability

The data that support the findings of this study are available from the corresponding author, upon reasonable request. The datasets presented in this study can be found in online repositories: https://github.com/lydia88success/data.
